# Case Report: Spinal cord herniation associated with ankylosing spondylitis

**DOI:** 10.3389/fsurg.2026.1818905

**Published:** 2026-10-16

**Authors:** Zekai Ke, Chunguang Duan

**Affiliations:** Department of Spine and Osteopathy, Shenzhen University General Hospital, Shenzhen, China

**Keywords:** ankylosing spondylitis, dural defect, kyphosis, pedicle subtraction osteotomy, spinal cord herniation

## Abstract

**Background:**

Spinal cord herniation (SCH) is a rare clinical entity characterized by the ventral displacement of the spinal cord through a dural defect. While often classified as idiopathic, SCH associated with ankylosing spondylitis (AS) is exceedingly rare. The pathogenesis in such cases remains unclear, and treatment strategies are not well standardized.

**Case presentation:**

Herein, we report a series of four patients with long-standing ankylosing spondylitis who presented with SCH between August 2018 and August 2021. All patients exhibited severe thoracolumbar kyphosis and restricted spinal mobility. Uniquely, the herniations occurred at the vertebral body level rather than the disc level, and one patient presented with rare multilevel herniations. Two patients underwent pedicle subtraction osteotomy (PSO) with spinal fusion. In both surgical cases, direct dural repair was avoided; stabilization was achieved through indirect cord relaxation via spinal column shortening. Postoperative imaging showed successful kyphosis correction and stabilization of the herniation.

**Conclusion:**

SCH in patients with AS is a distinct entity secondary to inflammatory and biomechanical changes. PSO with fusion is a viable surgical option that addresses both deformity and cord tethering through an indirect mechanism.

## Introduction

Spinal cord herniation (SCH) is a rare pathological condition defined by the protrusion of the spinal cord into the adjacent epidural space through a defect in the ventral dura mater. Since its initial description in 1974 (1), the majority of reported cases have been classified as idiopathic (ISCH), typically occurring in the thoracic spine (T2–T10) (2). Although ISCH has been widely discussed, SCH co-occurring with ankylosing spondylitis (AS) is an exceptionally rare phenomenon. In this study, we present a series of four patients with AS diagnosed with SCH, analyze their clinical characteristics, and evaluate the efficacy of pedicle subtraction osteotomy (PSO) as a tailored surgical solution. In this study, AS was diagnosed based on the Modified New York Criteria (1984) (3). The diagnosis of SCH was confirmed by MRI and CT showing ventral spinal cord kinking and protrusion through a dural defect.

## Case presentation

### Case 1

A 33-year-old male office worker with a 12-year history of AS (HLA-B27 positive) presented with progressive kyphosis. His medical history included long-term use of non-steroidal anti-inflammatory drugs (NSAIDs) with partial relief. There was no family history of spinal deformities or genetic disorders. Psychosocially, the patient reported significant anxiety regarding his worsening posture and its impact on his professional life. A neurological examination showed normal muscle strength (5/5) in all limbs, with no spasticity or bladder/bowel dysfunction. A preoperative MRI demonstrated a ventral deviation of the spinal cord at the vertebral body level (Figure 1A,B). The intervention involved T11 and L3 PSO. PSO achieved a kyphosis correction of 73° (Figure 1C). The preoperative sagittal vertical axis (SVA) could not be measured because a complete full-length lateral spine radiograph could not be obtained. The postoperative SVA was 150 mm. Blood loss was 600 mL, and the operative time was 460 min. The primary goal was deformity correction; no direct dural repair or cord manipulation was performed to avoid injury to the fragile, inflamed tissues. On his part, the patient expressed a high level of satisfaction with a significant improvement in spinal alignment and a complete relief of preoperative gait disturbance at the final follow-up (Table 1).

**Figure 1:**
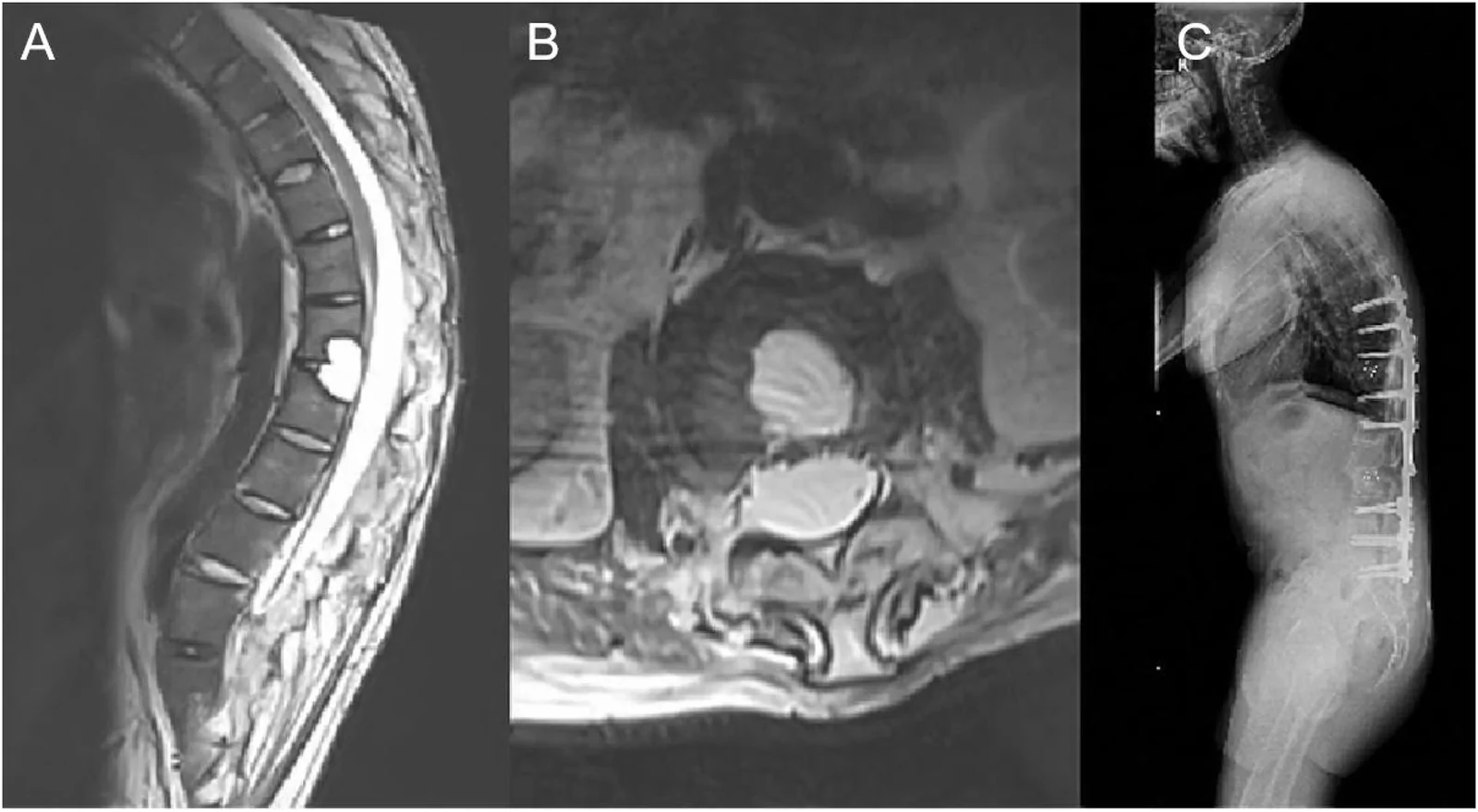
**(A)** Spinal cord herniation at the L1–L2 level in patient 1, associated with a bony defect and ventral displacement of the spinal cord along the spinal canal. **(B)** Herniation of the cauda equina and conus medullaris fibers into the bony defect of the L1 and L2 vertebral bodies. **(C)** Postoperative imaging demonstrating satisfactory correction of kyphosis.

**Table 1:** Timeline of episode of care for surgical patients (cases 1 and 2).

Time point	Milestone/clinical event
12–20 years preadmission	Onset of AS symptoms (back pain, stiffness); initiated NSAIDs and biological agents
2–3 years preadmission	Progressive thoracolumbar kyphosis; onset of mild lower limb numbness
Admission	Detailed physical examination; MRI and CT confirming spinal cord herniation
Day 5 of admission	Therapeutic intervention: PSO and long-segment fusion
Day 14 of admission	Postoperative mobilization; discharge
3–6 months postop	Follow-up imaging and neurological assessment

### Case 2

A 39-year-old manual laborer with a 15-year history of AS presented with severe sagittal imbalance. He had previously been treated with Adalimumab for 2 years. His family history was unremarkable. CT imaging revealed cord displacement into the T12 vertebral body (Figure 2A,B). We performed an L1 PSO. The decision to forgo direct dural intervention was a deliberate shift from standard idiopathic SCH protocols, justified by the patient's rigid spinal architecture and high risk of dural cerebrospinal fluid leak in the context of AS-related inflammation. A 33° correction of kyphosis was attained via PSO (Figure 2C). Although preoperative SVA evaluation was unfeasible due to the lack of complete full-length lateral radiographs, the postoperative SVA was measured at 154 mm. The procedure involved an estimated blood loss of 500 mL and an operative time of 260 min. The patient experienced transient postoperative weakness, but he completely recovered. There was left lower limb muscle atrophy (quadriceps and tibialis anterior), with strength graded at 4/5; bowel and bladder functions were intact (Table 1).

**Figure 2:**
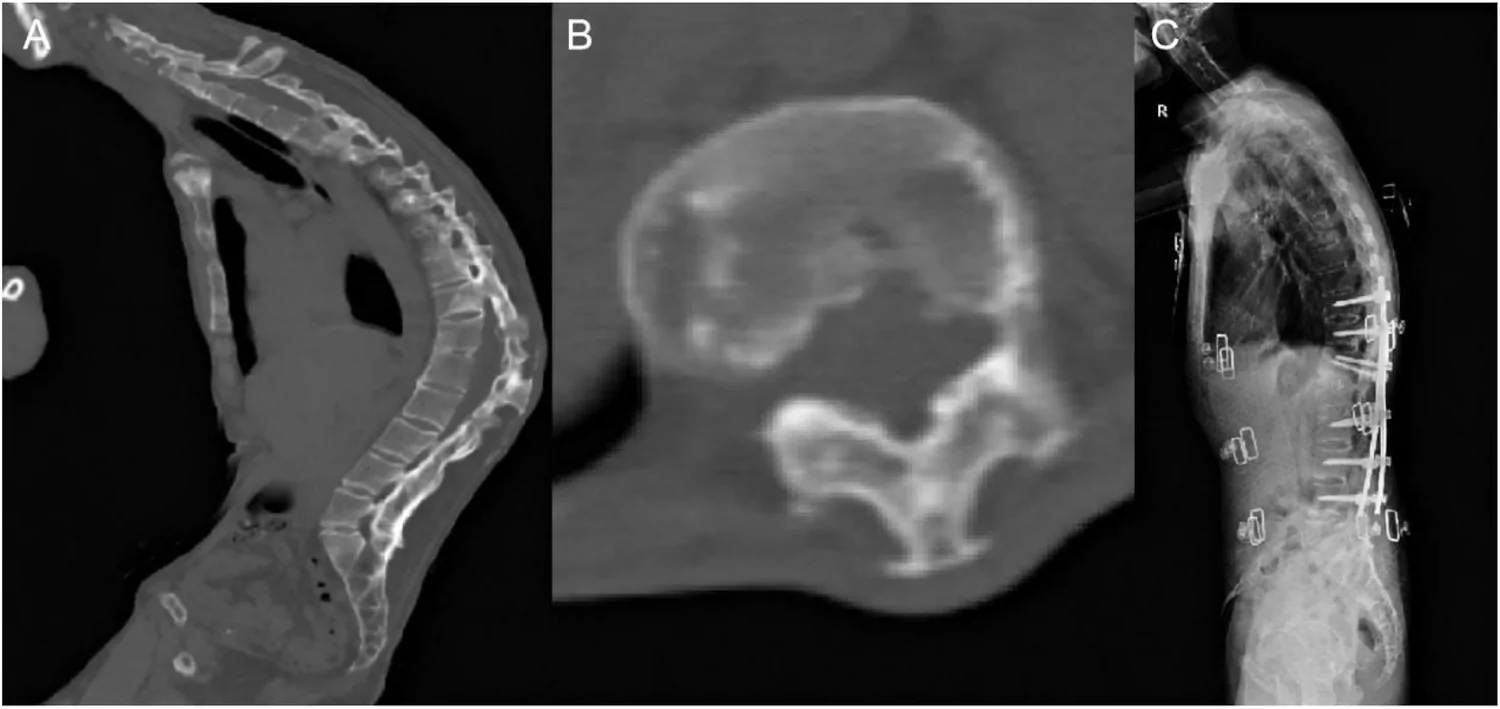
**(A)** Sagittal CT image of patient 2 demonstrating spinal cord herniation. **(B)** Axial CT image at the T12 level showing spinal cord herniation. **(C)** Postoperative imaging demonstrating satisfactory deformity correction.

### Case 3

A 36-year-old man presented with a 20-year history of back pain and stiffness. An MRI revealed an anterior kink and herniation of the spinal cord at the L1 and L2 levels (Figure 3). The patient declined surgical intervention and was therefore managed conservatively.

**Figure 3:**
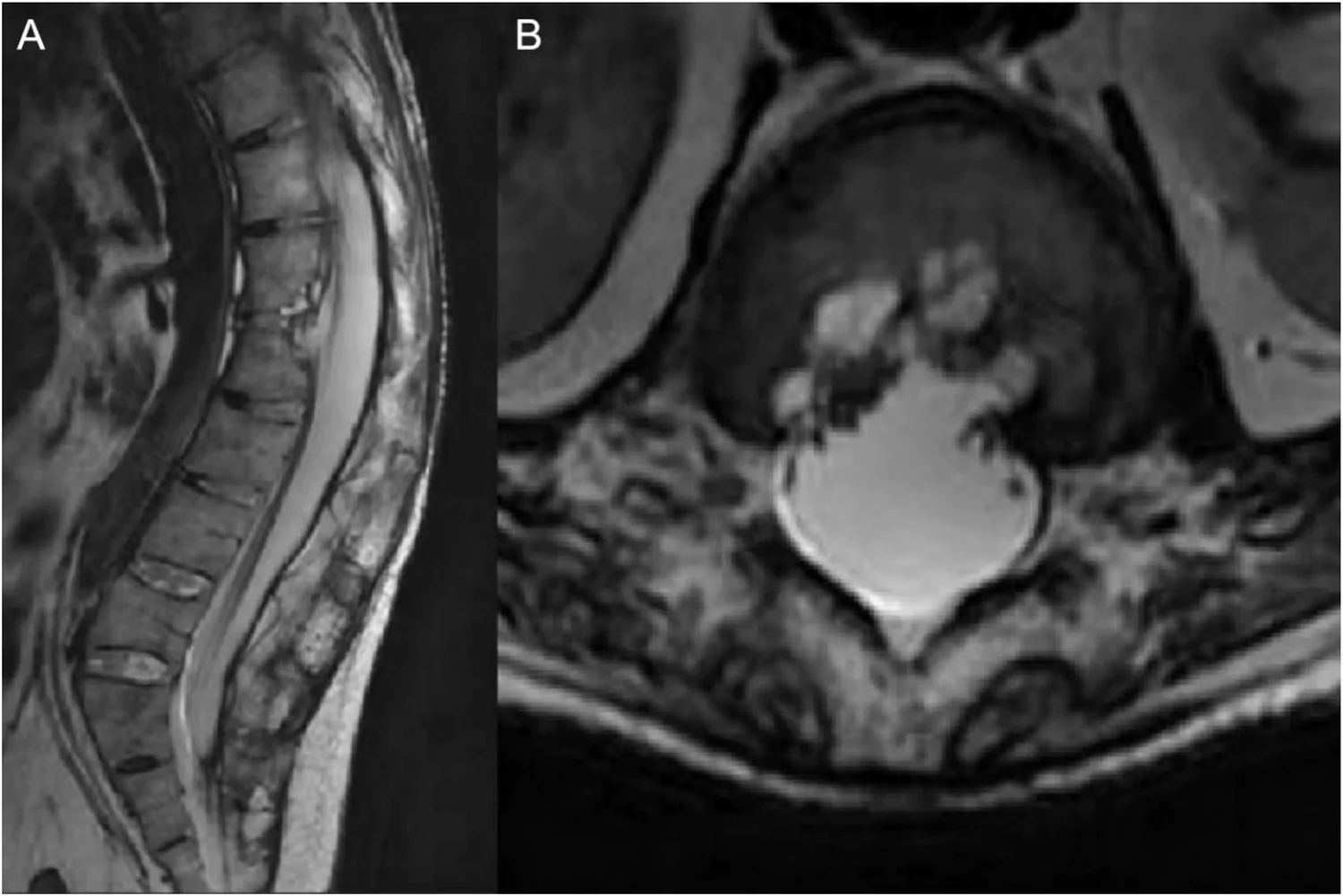
**(A,B)** Sagittal and axial images, respectively, of patient 3 demonstrating spinal cord herniation at the L1–L2 level.

### Case 4

A 51-year-old woman presented with worsening kyphosis. An MRI revealed a rare multilevel spinal cord herniation at T11/T12 and T12/L1 (Figure 4). The patient opted against surgical treatment.

**Figure 4:**
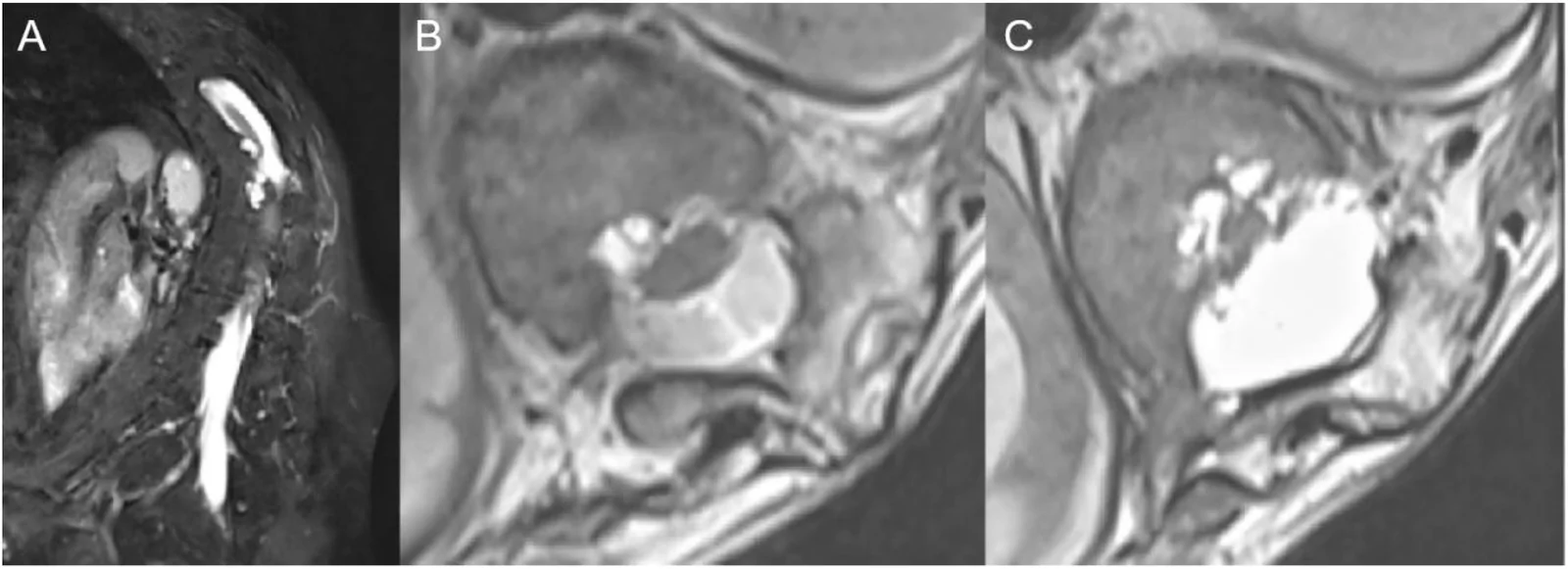
**(A)** Sagittal image demonstrating spinal cord herniation at the T11/T12 and T12/L1 levels. **(B)** Axial image at the T11/T12 level showing spinal cord herniation. **(C)** Axial image at the T12/L1 level showing spinal cord herniation.

## Discussion

### Pathogenesis: the role of inflammation and biomechanics

The cases presented in this series differ significantly from typical ISCH. First, the herniations occurred in the thoracolumbar region at the vertebral body level, rather than the midthoracic disc level (2). We hypothesize that chronic rheumatic inflammation at the dural interface leads to arachnoid adhesions and potential dural fragility. When combined with the tensile forces of severe kyphosis, these factors facilitate a ventral dural defect. This supports the theory that SCH in AS is an acquired, disease-induced phenomenon.

### Surgical management: the rationale for PSO

The treatment of typical ISCH usually involves direct repair (4–6) or widening of the dural defect (7). However, in patients with AS, the herniation is complicated by rigid deformity and inflamed, fragile tissues. We utilized PSO for two primary reasons.

Deformity Correction: Addressing the sagittal imbalance.

Indirect Cord Relaxation: By shortening the posterior column, PSO shortens the spinal canal. This reduces the anterior tension on the spinal cord, allowing it to “relax” away from the dural defect without requiring high-risk direct manipulation of the cord or the fragile ventral dura.

### Tailored surgical strategy for AS-related SCH

When encountering SCH in AS, we recommend the following tailored steps:

1. Level Identification: Focus on the vertebral body level in the thoracolumbar transition.
2. Posterior Column Shortening: Use PSO to simultaneously correct deformity and reduce cord tension.
3. Avoidance of Direct Dural Repair: Given the tissue fragility and technical difficulty in rigid kyphosis, indirect stabilization via column shortening is preferred.
4. Long-Segment Fusion: To maintain alignment and prevent recurrent biomechanical stress.

## Strengths and limitations

To our knowledge, this is the first case series to describe the clinical and radiographic features of SCH in patients with long-standing AS, which may contribute to the limited literature on this rare association. However, several limitations should be acknowledged. First, this is a retrospective, single-center study with a small sample size, which limits the generalizability of our findings and the proposed PSO strategy; thus, further multicenter validation is warranted. Second, owing to its retrospective nature, objective parameters of systemic AS disease activity (such as BASDAI or ASDAS scores) were not systematically available. Third, direct intraoperative or histopathological confirmation of the dural defect was not performed, as direct dural repair was avoided to minimize surgical risk, operative time, and blood loss. Last, given the descriptive nature of this case series and the absence of a control group, a definitive causal relationship between AS and SCH cannot be established, and alternative etiologies for dural thinning or vertebral body erosion cannot be completely excluded.

## Conclusion

SCH in patients with AS is a distinct entity. PSO serves as an effective treatment option, simultaneously correcting the deformity and stabilizing the spinal cord through indirect relaxation.

## Data Availability

The original contributions presented in the study are included in the article/Supplementary Material, and further inquiries can be directed to the corresponding author.
